# Mechanical and free living comparisons of four generations of the Actigraph activity monitor

**DOI:** 10.1186/1479-5868-9-113

**Published:** 2012-09-12

**Authors:** Mathias Ried-Larsen, Jan Christian Brønd, Søren Brage, Bjørge Herman Hansen, May Grydeland, Lars Bo Andersen, Niels Christian Møller

**Affiliations:** 1 University of Southern Denmark, Institute of Sports Science and Clinical Biomechanics, Centre of Research in Childhood Health, Campusvej, Odense M, 55 5230, Denmark; 2 MRC Epidemiology Unit, Institute of Metabolic Science, Cambridge, UK; 3 Department of Sports Medicine, Norwegian School of Sport Sciences, Oslo, Norway

**Keywords:** Accelerometry, Physical activity assessment, Physical activity intensity, Mechanical testing, Free living

## Abstract

**Background:**

More studies include multiple generations of the Actigraph activity monitor. So far no studies have compared the output including the newest generation and investigated the impact on the output of the activity monitor when enabling the low frequency extension (LFE) option. The aims were to study the responses of four generations (AM7164, GT1M, GT3X and GT3X+) of the Actigraph activity monitor in a mechanical setup and a free living environment with and without enabling the LFE option.

**Methods:**

The monitors were oscillated in a mechanical setup using two radii in the frequency range 0.25-3.0 Hz. Following the mechanical study a convenience sample (N = 20) wore three monitors (one AM7164 and two GT3X) for 24 hours.

**Results:**

The AM7164 differed from the newer generations across frequencies (p < 0.05) in the mechanical setup. The AM7164 produced a higher output at the lower and at the highest intensities, whereas the output was lower at the middle intensities in the mid-range compared to the newer generations. The LFE option decreased the differences at the lower frequencies, but increased differences at the higher. In free living, the mean physical activity level (PA) of the GT3X was 18 counts per minute (CPM) (8%) lower compared to the AM7164 (p < 0.001). Time spent in sedentary intensity was 26.6 minutes (95% CI 15.6 to 35.3) higher when assessed by the GT3X compared to the AM7164 (p < 0.001). Time spend in light and vigorous PA were 23.3 minutes (95% CI 31.8 to 14.8) and 11.7 minutes (95% CI 2.8 to 0.7) lower when assessed by the GT3X compared to the AM7164 (p < 0.05). When enabling the LFE the differences in the sedentary and light PA intensity (<333 counts*10 sec^-1^) were attenuated (p > 0.05 for differences between generations) thus attenuated the difference in mean PA (p > 0.05) when the LFE option was enabled. However, it did not attenuate the difference in time spend in vigorous PA and it introduced a difference in time spend in moderate PA (+ 3.0 min (95% CI 0.4 to 5.6)) between the generations.

**Conclusion:**

We observed significant differences between the AM7164 and the newer Actigraph GT-generations (GT1M, GT3X and GT3X+) in a mechanical setup and in free-living. Enabling the LFE option attenuated the differences in mean PA completely, but induced a bias in the moderate PA intensities.

## Introduction

The Actigraph (Pensacola, FL) activity monitors have been, and are, widely used in large population studies for the objective assessment of physical activity (PA) [1-4]. During the past decades, activity monitors have developed, with changes made to hardware and firmware.

Several studies have reported tracking and secular trends of PA behaviors [1,3,5]; such analyses are very sensitive to systematic measurement error. Thus, comparing longitudinal accelerometer data retrospectively requires thorough investigations on inter-generation activity monitor output to ensure comparability of instruments and thereby ensure unbiased population-based PA estimates over time. As the types of studies are used for generating public health policies, biased estimates would have severe consequences.

Studies have compared the first two generations of Actigraph activity monitor; the AM7164 and the GT1M in mechanical [6], laboratory setups [7-9], and free-living conditions [10] and to our knowledge only one study have included the third generation GT3X in comparison to the GT1M [11]. Observations made so far suggest that inter-generation differences do seem to exist at certain intensities between the older generation AM7164 and the newer GT1M; however none of the studies have to our knowledge included all four generations; AM7164, GT1M, GT3X and the latest GT3X + .

There is an interest among researchers on sedentary behavior as a predictor of disease outcomes [12,13]. So far no studies have assessed the similarities of the oldest and the newest Actigraph monitors at the low intensities equivalent to light PA and sedentary behaviors. With the GT3X, Actigraph introduced the low frequency extension (LFE) option. The LFE option is designed to increase the sensitivity by extending; *“…the lower end (baseband) of the filter cutoff, effectively expanding the bandwidth of the accumulated data”*[14]. However, the LFE option can be applied to the GT1M monitor. An adjustment of the filter at the lower end of the frequency range would potentially not only affect the interpretation of important health parameters, such as time spend on sedentary behavior, but also impact on wear/non-wear classification and thereby on most accelerometer derived PA parameters. This would challenge standardization of measures within studies using different generations of monitors over time, and between study comparisons and lead to erroneous conclusions in studies assessing the effect of sedentary behavior on health. The effect of the LFE option on the similarity of the output between the different generations of monitors has not yet been explored.

Therefore the aims of the study are to 1) investigate the vertical axis responses of the Actigraph activity monitor generations AM7164, GT1M, GT3X and GT3X + throughout a wide range of accelerations using a mechanical setup, 2) investigate whether enabling the LFE option affects the similarity of the vertical axis output between the AM7164 and the GT3X in a mechanical setup, and 3) to confirm observations made in the mechanical setup in a free living scenario.

## Methods

### Mechanical setup

#### Equipment

Four generations of the Actigraph activity monitors were used; model AM7164 (n = 22), model GT1M (n = 22), model GT3X (n = 22), and the model GT3X + (n = 22). Model AM7164 is uniaxial and it is designed to measure movement in one (usually the vertical) direction, whereas the GT1M measures in two orthogonal directions, and the GT3X and GT3X + measures in three orthogonal directions. The AM7164, GT1M and GT3X are usually set up to sample in predefined epoch lengths, with on-board filtering. The GT3X + has a configurable sampling frequency during initialization of the monitor, ranging from 30 to 100 Hz in 10 Hz increments, with different options for post-sampling filtering (and analysis epoch lengths) performed by the Actilife software. The sampling frequency in the GT3X + was set at 30 Hz. Technical specifications are described in detail elsewhere [15]. The AM7164 were borrowed from two different study centers in order to account for potential inter-batch differences [16,17]. AM7164 activity monitors were purchased from 2003 to 2005 and GT3X activity monitors from 2009–2010, GT1M activity monitors were purchased in 2008 and GT3X + activity monitors in 2011. Prior to the mechanical testing, the AM7164 monitors were calibrated using the Actigraph manufactured calibrator (Model CAL71).

AM7164 activity monitors were initialized and downloaded via a serial port interface using a DOS-based program (RUI24, v. 2.13B, Computer Science and Applications INC.) and the newer monitor generations were initialized and downloaded via a USB interface using the Actilife software (v. 5.8.3, the Actigraph) (Table 1).

**Table 1:** Description of equipment and software

**Model**	**Dimensions (cm)**	**Band pass (Hz)**	**Dynamic range (g)**	**Sampling frequency (Hz)**	**Accelerometer**	**Firm ware**
***AM7164***	5.1 x 4.1 x 1.5	0.21-2.28	0.05-2.13	10 Hz	Piezoelectric	v. 2.2
***GT1M***	3.8 x 3.7 x 1.8	0.25-2.5	0.05-2.5	30 Hz	Solid state (MEMS)	v. 7.5.0
***GT3X***	3.8 x 3.7 x 1.8	0.25-2.5	± 3	30 Hz	Solid state (MEMS)	v. 4.4.0
***GT3X+***	4.6 x 3.3 x 1.5	0.25-2.5	± 6	30-100 Hz	Solid state (MEMS)	v. 2.1.0

Micro electro-mechanical system (MEMS).

### Experimental setup

**Figure 1:**
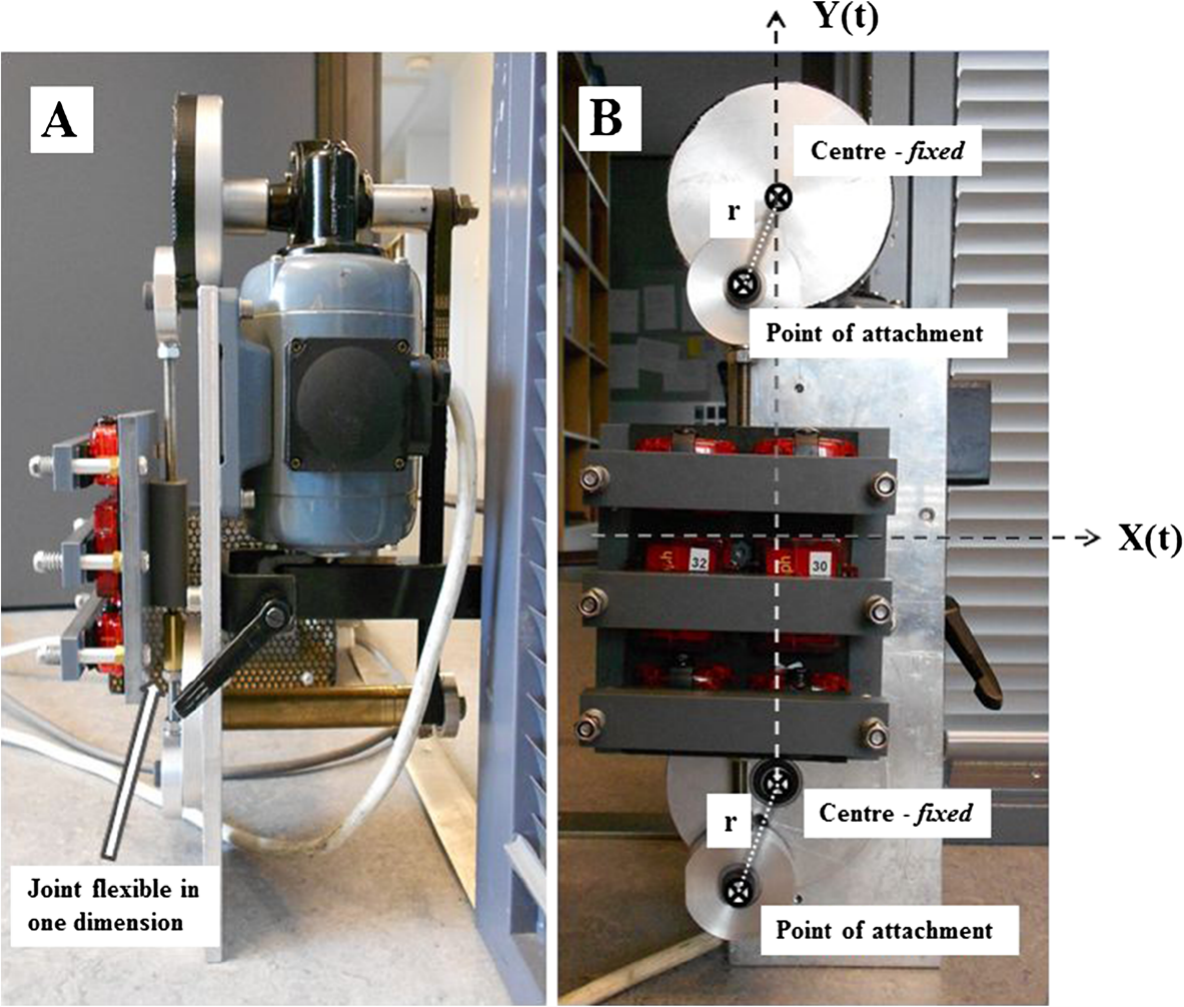
**Calibration machine used in the laboratory from the side (A) and from the front (B) with abscissa X(t) and ordinate, Y(t) and the radius* (r)*.**

In order to produce accelerations, we used a custom made mechanical setup, described in detail elsewhere [18]. It consists of two rotational wheels rotating in the same plane at a constant (but operator controlled) angular velocity (ω) (rad·s^-1^) (Figure 1). The wheels are connected with a connection rod (CR) and driven by an electric motor. The CR is attached away from the center of the rotational wheels. The monitors were firmly secured on a plate attached to the CR. This setup produces positive and negative accelerations in a single plane with maximal displacement along any one axis equal to two times the length of the radius of oscillation (r) from the centre of the rotational wheels to the connection point of the CR. Radius (r) is adjustable in three different lengths (22.0, 35.5 mm, and 49.0 mm) but only r = 22 mm and r = 49 mm were used in this study, as they represent the extreme ends of normal center of gravity displacement during gait [19]. 

The frequency of oscillation (f) (s^-1^) is directly related to ω as:

(1) $$\omega =\pi \cdot f^{2}$$

The acceleration (A) is given as:

(2) $$A=8\cdot \pi \cdot r\cdot f^{2}$$

In order to exclude a possible effect of the monitor position on the plate, a pre-protocol location experiment with three GT3X accelerometers was performed. Each monitor was placed on all six possible locations on the plate. No differences between locations were observed in the frequency range up to 3 Hz (data not shown).

### Procedures and data reduction

The trial was repeated using both a ‘short’ (r = 22 mm) and a ‘long’ (r = 49 mm) radius. The epoch was set to one second to ensure a high resolution of the data. Even though the upper band limit of the accelerometers is 2.5 Hz, we examined the response up to a frequency roof of 3 Hz, as some human movement is observed to be higher than the upper band limit [19,20]. In order to produce high resolution response curves, measurements were obtained with 0.05 Hz increments from 0.2 to 0.4 Hz, and with 0.1 Hz increments from 0.4 to 3.0 Hz. This yielded a total of 30 frequencies and 1800 data points per monitor. For the second aim, the data from GT3X + models were re-processed using the Actilife software with the LFE option enabled. To ensure that the results were not affected by potential resonance in the mechanical setup, Fast Fourier Transform analyses were conducted using 30 Hz raw waveform data from the GT3X+. No major resonance areas were identified (data not shown).

### Free living study

A convenience sample (N = 20, mean age (sd) 37.8 (8) years) wore a AM7164, GT3X (without the LFE option enabled, GT3Xa) and a GT3X (with the LFE option enabled, GT3Xb) for 24 hours (only removed during showering or swimming) on the right hip in one belt. Epoch time was set to 10 seconds. All AM7164 monitors were accepted by the Actigraph manufactured calibrator (Model CAL71) pre and post study. The monitors were placed in random position order in the belt. Time spent at different intensities defined as; sedentary (<17 counts*10 sec^-1^) [21], light (, 18–333 counts*10 sec^-1^), moderate (334–1000 counts*10 sec^-1^) [22], moderate and vigorous (>333 counts*10 sec^-1^) [22-25] and vigorous (>1000 counts*10 sec^-1^) [23-25] PA. As cut-points vary greatly across studies, we chose the cut-points to approximate variety of typically used and validated cut-points. The partitioning of data was performed using a custom made software (Propero 1.018). The study was approved by the Regional Scientific Ethical Committee for Southern Denmark (Project ID: S-20122000-100) and data was collected according the Helsinki declaration.

### Statistics and data reduction

Assessment of inter-generation differences within each frequency, radius, and LFE option was done using a one-way ANOVA allowing for clustering on monitor level (in the mechanical setup) or subject level (in free living) (using the *vce cluster* option).

All p-values derived from the mechanical setup and free living were Bonferroni adjusted to account for multiple comparisons between generations (mechanical setup and free living) and within each frequency (mechanical setup only). Statistical significance level was inferred at 0.0125 for comparisons between all four generations within the frequencies and at 0.017 for comparisons between three generations. Inter generation coefficients of variation were computed within each frequency. All analyses were conducted using STATA SE 11.1. (StataCorp, TX, USA).

## Results

### Mechanical setup

#### Comparisons between Actigraph generations (LFE option disabled)

##### Short radius

**Figure 2:**
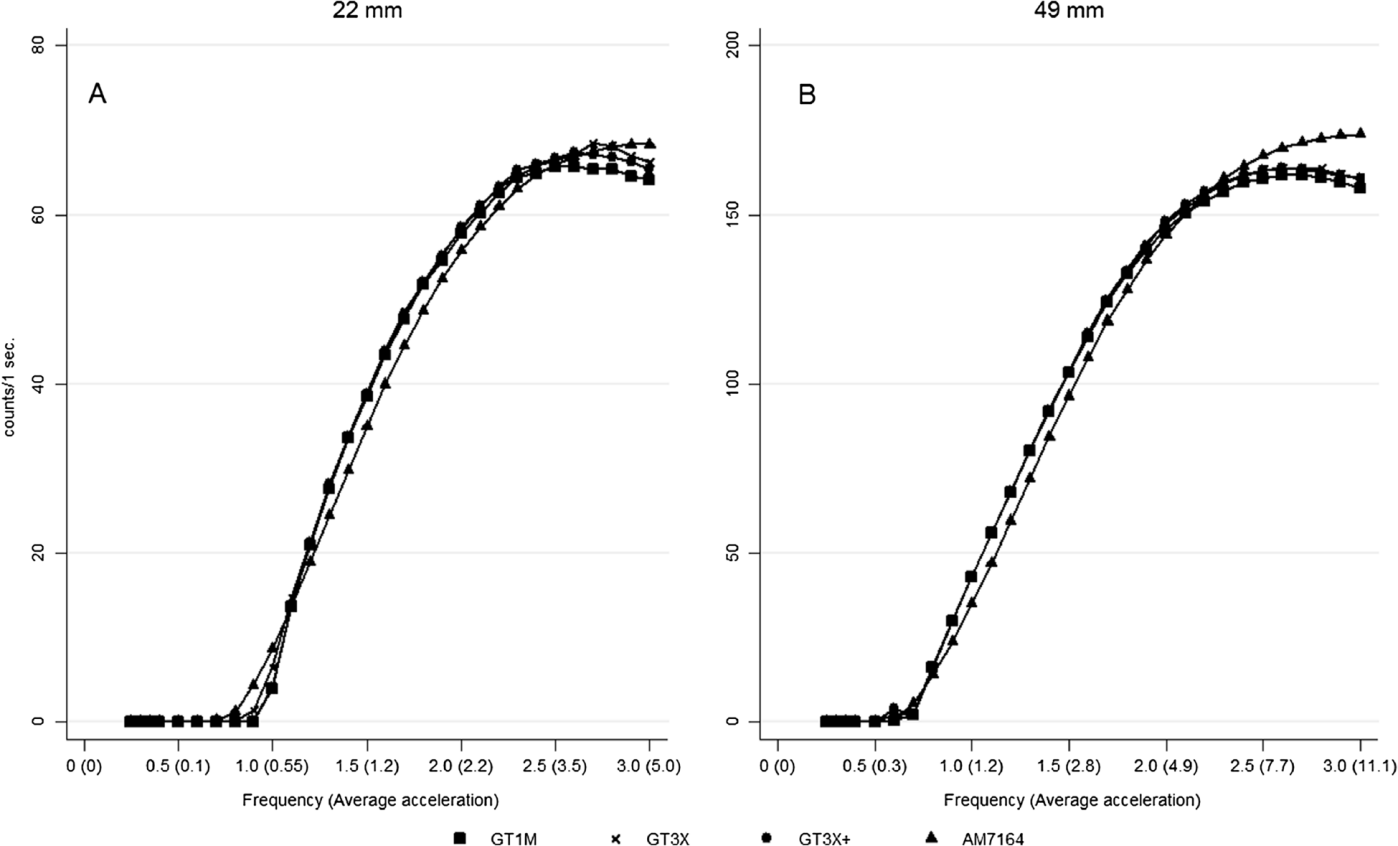
**The figure describes the activity monitor output as a function of the frequency at both short radius (22 mm) of oscillation (A) and long radius (49 mm) of oscillation (B), with the Low Frequency Extension (LFE) option disabled.** Frequency is expressed as s^-1^ and the average acceleration in ms^-2^. The results are presented as means and the standard error of the mean (SEM). The scales differ between radii of oscillation.

Data from all monitors (N = 84) were successfully downloaded and included in the analyses. Figure 2 displays the monitor outputs using r = 22 mm (Figure 2A) without the LFE option enabled. All monitors displayed a zero count output below 0.7 Hz. (0.28 m/s^2^). The shape of the AM7164 response curve was different compared to those of the GT1M, GT3X and the GT3X+, generating significantly higher outputs at 0.8-1.0 Hz. (Δ_min-max_ ≈ 1–4 counts·sec^-1^, (p < 0.001) and 2.8-3.0 Hz (Δ ≈ 3 counts·sec^-1^, p < 0.001). Significantly lower output from AM7164 was observed at frequencies 1.2-2.2 Hz (Δ_min-max_ ≈ 2-6 counts·sec^-1^, p < 0.001), generating the highest difference at 1.5 Hz. The shape of the GT1M, GT3X and GT3X + response curves were very similar, even though the GT1M output was significantly lower at frequencies 2.7-3.0 Hz. (p < 0.001) and the GT3X was significantly higher at 1.0 Hz. (p < 0.001). The AM7164 curve crossed the other curves at approx. 15 counts·sec^-1^ (≈900 counts per minute (CPM) and again at approx. 66 counts·sec^-1^ (≈4000 CPM) at this radius. Standard errors of the mean (SEM) ranged between 0.0004 counts·sec^-1^ at 0.7 Hz (the lowest intensity with a detectable output) and 0.2 counts·sec^-1^ at the highest intensities (<2.5-3.0 Hz). This did not differ between generations.

##### Long radius

Figure 2 displays the outputs using r = 49 mm (Figure 2B). All monitors displayed a zero count output below 0.5 Hz. (0.30 m/s^2^). As observed for the 22 mm radius, the shape of the AM7164 response curve at r = 49 mm differed from the GT1M, GT3X and GT3X+, with significantly higher outputs at 0.7 Hz (Δ ≈ 3.3 counts·sec^-1^, p < 0.017) and 2.5-3.0 Hz (Δ_min-max_ ≈ 5-14 counts·sec^-1^, p < 0.017) and a significantly lower output at frequencies 0.8-2.0 Hz (Δ_min-max_ ≈ 2.5-9 counts·sec^-1^, p < 0.001) with the highest difference at 1.1 Hz and the lowest at 0.8 Hz (p < 0.001). There was no significant difference between the AM7164 and the GT1M at 0.6 Hz. (p > 0.017) but the GT3X and GT3X + output was significantly higher than the two older generations (Δ ≈ 2.5 counts·sec^-1^, p < 0.017). The response curves of GT3X and GT3X + monitors were of similar shape (no differences between outputs (p > 0.017)). The GT1M curve shape, however, was different from the response curve of the GT3X and the GT3X + at the higher frequencies resulting in significantly lower outputs from 1.8-3.0 Hz (Δ_min-max_ ≈ 1-3 counts·sec^-1^, p < 0.017). The AM7164 curve crossed the other curves at approx. 150 counts·sec^-1^ (≈9000 CPM).

SEM ranged between 0.001 counts·sec^-1^ at 0.5 Hz (the lowest intensity with a detectable output) and 0.2 counts·sec^-1^ at 2 Hz. This did not differ between generations and did not change above 2 Hz for the newer generations. However, the AM7164 displayed SEM ranging from 0.25 to 0.6 counts·sec^-1^ at frequencies 2.1-3.0 Hz.

#### Comparisons between the AM7164 and the GT3X + (LFE option enabled)

**Figure 3:**
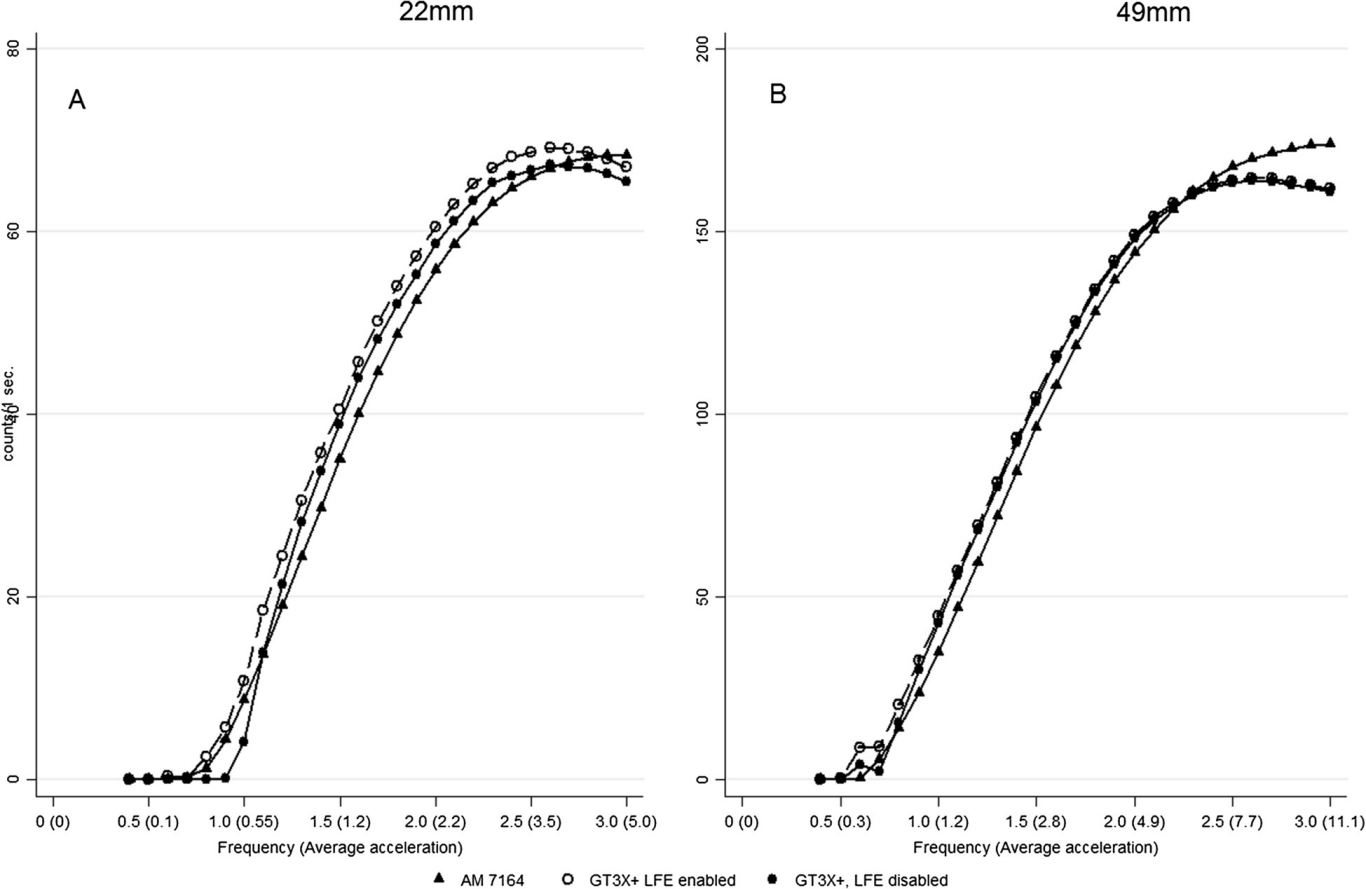
**The figure describes the AM7164 and the GT3X + with and without the Low Frequency Extension (LFE) option disabled accelerometer.** The plot is the output as a function of the frequency at short radius (22 mm) of oscillation (**A**) and long radius (49 mm) of oscillation (**B**). Frequency is expressed as s^-1^ and the average acceleration in ms^-2^. The results are presented as means. The scales differ between radii of oscillation.

Figure 3 describes the AM7164 and the GT3X + activity counts at r = 22 mm, and without the LFE option enabled (Figure 3A). Enabling the LFE option shifted the GT3X + curve to the left. This resulted in significant higher outputs at all frequencies compared to GT3X + activity counts with the LFE disabled (p < 0.017). The largest differences were observed below 1.2 Hz (Δ_min-max_ ≈ 0.1-5 counts·sec^-1^, p < 0.017) with the largest difference observed at 1.1 Hz and the smallest at 0.6 Hz. At the higher frequencies the differences were constant at around 2 counts·sec^-1^ (95% CI 1.95-1.97, p < 0.017).

The GT3X + activity counts with the LFE option enabled was significantly higher compared to the AM7164 at 0.6 Hz (Δ ≈ 0.4 counts·sec^-1^, p < 0.017)) and from 0.8- 2.5 Hz (Δ_min-max_ ≈ 1-6 counts·sec^-1^, p < 0.017). The smallest difference was observed at 0.8 Hz and the largest at 1.5 Hz. The AM7164 and the GT3X + activity counts at 49 mm with and without the LFE option enabled are displayed in Figure 3B. As for r = 22 mm, enabling the LFE option resulted in a shift of the curve to the left resulting in a significant higher output at all frequencies compared to the GT3X + with the LFE option disabled (p < 0.017). The largest differences were observed below 1.0 Hz (Δ_min-max_ ≈ 0.5-2.5 counts·sec^-1^, p < 0.017). The smallest difference was observed at 0.4 Hz and the largest at 0.9 Hz. At the higher frequencies the differences were constant at around 1 counts·sec^-1^ (95% CI: 0.97-1.01 counts·sec^-1^, p < 0.017). Enabling the LFE did not change SEM.

#### Free living

Data was successfully downloaded from all subjects (N = 20). Mean wear time (sd) was 23.98 (0.02) hours. As only ten participants reported removal of the monitors during swimming and showering (mean non-wear 6.1 min), it was decided not to exclude any data. Table 2 shows the mean values of the output for all activity monitors and settings. Table 3 shows the absolute differences between the activity monitors. 

**Table 2:** Mean free-living physical activity level and intensities

	**AM7164**	**GT3X**	**GT3X (LFE)**
*Mean PA (counts per minute)*	219 (122)	198 (111)	216 (122)
*Sedentary (min)*	1198.9 (63.0)	1224.5 (54.4)	1196.4 (61.8)
*Light (min)*	195.7 (48.3)	172.1 (39.7)	197.6 (46.0)
*Moderate (min)*	37.7 (19.4)	38.0 (19.2)	40.7 (22.0)
*Vigorous (min)*	6.3 (11.0)	4.5 (9.7)	4.9 (10.0)
*Moderate-and-vigorous (min)*	43.9 (28.2)	42.5 (26.4)	45.6 (29.8)

Data are means (SD).

**Table 3:** Absolute inter-generation differences during free-living

	**GT3X (LFE disabled) vs. AM7164**	**GT3X (LFE enabled) vs. AM7164**
*Mean PA (counts per minute)*	−18 (−28 to −7)†	−3 (−6 to 10)
*Sedentary (min)*	26.6 (15.6 to 35.3)†	−2.5 (−11.0 to 6.1)
*Light (min)*	−23.3 (−31.8 to −14.8)†	2.2 (−6.6 to 10.9)
*Moderate (min)*	0.3 (−2.3 to 2.9)	3.0 (0.4 to 5.6)
*Vigorous (min)*	−1.7 (−2.8 to −0.7) †	−1.3 (−2.4 to −0.2)*
*Moderate-and-vigorous (min)*	−1.4 (−4.0 to 1.2)	1.7 (−0.7 to 4.0)

Data are mean differences (95% CI).

The mean CPM for the GT3X without the LFE enabled was 18 CPM (8%) lower compared to the AM7164 (p < 0.001). Compared to the AM7164, the GT3X (LFE disabled) produced 1.7 min (28%) more time in vigorous PA and 26.6 min (2%) less time in sedentary and 23.3 min (12%) less light PA (p < 0.001). No differences between the monitors were observed in moderate and moderate-and-vigorous PA (p > 0.05).

Enabling the LFE attenuated the differences for CPM, sedentary time and light PA. However, there was still a significant difference between the AM7164 and the GT3X (LFE enabled) in vigorous PA (p < 0.05). Furthermore, when enabling the LFE, the GT3X produced 3.0 min (7%) more time in moderate PA compared to the AM7164 (p < 0.05).

We performed a post hoc analysis where the sample was classified according to the UK PA guidelines [26]. It is recommended that adults should accumulate at least 150 minutes of moderate-and-vigorous PA per week, e.g. 30 minutes per day five times a week. Based on the free-living data and using the 30 minute per day cut-point, fifteen persons fulfilled the guidelines on the basis of data from the GT3X (LFE disabled) and thirteen when the classification was based on the AM7164. For the two persons differently classified, the time spend in moderate-and-vigorous intensity PA was 25.3 and 22.5 minutes when assessed using the GT3X and 35.8 and 31.3 minutes when assed using the AM7164. The equivalent time spent in moderate-and-vigorous assessed by the GT3X with the LFE enabled were 28.8 and 25.5 minutes, respectively. We reassessed the classification using a 500 counts*10 sec^-1^ (~3000 CPM) cut point for moderate-and-vigorous PA. This did not change the difference in classification.

## Discussion

The main findings of this study are; 1) significant differences exist between the older piezoelectric-based monitors and newer micro electro-mechanical system (MEMS) based monitors, throughout a wide range of frequencies and different radii of oscillation in a mechanical setup and; 2) the direction of the differences observed in the mechanical setup were confirmed in a free living scenario resulting in differences in mean PA level and across PA intensities and 3) enabling the LFE did not increase similarities in the mechanical setup but attenuated the differences in mean PA between the AM7164 and GT3X in the free-living setup due to increased similarities in sedentary time and light PA.

As observed in the mechanical setup, the differences in free living were “non-systematic” throughout the intensity interval. This resulted in an eight percent lower mean PA of the GT3X compared to the AM7164 in free living. Enabling the LFE option attenuated the difference between generations in mean CPM due to an attenuation of differences in time spent in sedentary and light PA. Enabling the LFE option did not attenuate the differences observed in time spent on vigorous PA, but induced a larger difference in time spent on moderate PA.

We observed that the differences between monitors affected how persons were classified according the current PA guidelines. This could introduce biased estimates on population level when estimating the number of persons complying with guidelines. This post hoc analysis should be interpreted with caution as the sample was small and the calculation was based on a single day of measurements. There are currently no recommendations on non-specific sedentary time.

### Differences between generations

Response curves of AM7164 and GT1M are broadly comparable to previous observations from studies using sinusoidal oscillations [6,18]. Rothney et al. observed that the AM7164 yielded a higher output and a steeper slope (higher gain with increasing acceleration) compared to the GT1M monitor in the low frequency range (<1000 CPM) indicating a lower sensitivity of the GT1M [6]. Our observations support this notion for all newer MEMS-based Actigraph activity monitors at the shorter radius. At the longer radius this was only the case for the GT1M, indicating that either the MEMS accelerometer and/or the filtering approach differs between the newer generations. The difference between the GT1M and the GT3X was confirmed post hoc, by rerunning six GT3X randomly chosen from a new batch of monitors. We did not rerun the GT1Ms as our observations were comparable to previous observations.

Only a few studies have compared different generation of the Actigraph activity monitors in humans, showing varying results. John et al. did not find any differences between the AM7164 and the GT1M [7], whereas Fudge et al., observed higher output of the GT1M compared to the AM7164 [7,9], using a similar sample and protocol. During self-paced locomotion at three self-selected speeds (mean (SD)); slow 0.7 (0.22) m·s^-1^, medium 1.3 (0.17) m·s^-1^ and fast 2.1 (0.61) m·s^-1^. Kozey et al. found a 2.7% higher output of the GT1M, compared to the AM7164 across all speeds (p < 0.05). Analyzing the result by speed, the GT1M only provided significantly higher outputs at the medium speed (5.3%, p < 0.05) [8]. The two latter observations are in accordance to our observations in the moderate and higher intensities. Only one study has so far compared the GT1M to the GT3X; 50 healthy participants performed a treadmill test at four medium to high intensity speeds (4.8, 6.4, 9.7 and 12 km·h^-1^) [11]. As in the mechanical setup, they did not observe any differences between the outputs on the vertical axis between the two generations in the mid-range intensities.

To our knowledge only one study has compared the AM7164 with the GT1M monitors during free-living conditions. Corder et al. found that the GT1M yielded a 9% lower output (p < 0.05) compared to the AM7164, (LOA; -36% to 23%) and the difference between the monitors increased with activity level in Indian adolescents. This did not translate into observable differences in time spent at moderate and high intensities [10]. The difference in mean CPM was similar compared to the difference in our free living study. In contrast we also observed significant differences in time spent in moderate and vigorous PA. In the mechanical setup the differences between generations seemed to depend on the radii and thus the magnitude of displacement in free living. As the size and the direction of the inter-generation bias is intensity dependent, the absolute differences during free-living conditions would depend on the time spent at certain intensities and the magnitude of displacement during locomotion. The differences between monitor generations may therefore be population-specific. Therefore, the difference could be ascribed the population (adult compared to child sample) or differences in activity types.

More free-living studies on different subpopulations are needed to interpret the impact of the differences observed in the mechanical setup in free-living. These studies would be most informative if waveform acceleration data were collected alongside epoch-level data with analogue Actigraphs; raw data may then be exposed to the same mathematical operations currently imbedded in firmware to produce epoch-level count data.

### Low frequency extension option

Enabling the LFE option did alter the response curve of the GT3X and GT3X + at all frequencies at both radii. This increased the count output in the low frequency range and did in fact decrease the difference between the AM7164 and the GT3X + in the low frequency range at the short radius as described by Actigraph. This *decreased* the difference between the monitor outputs at the lower frequencies. However, the upward shift of the curve *increased* the difference between the AM7164 and the GT3X + at the higher frequencies. In free living enabling the LFE option attenuated the differences of the mean PA level and time spend sedentary in our free living study. Therefore, it could be advisable to use this option in future studies. As the direction of the bias introduced by the LFE option observed in the mechanical setup was confirmed in free living, thus increased the differences at the moderate intensities, using the LFE option to the decrease the inter-generation differences, should be done with caution. This observation needs to be confirmed in other samples.

In summery we observed differences between the old and newer generations of Actigraph monitors throughout a wide range of frequencies at different radii and that the absolute differences depend on both frequency and amplitude. The pattern observed in the mechanical setup was confirmed in free living. When enabling the LFE option, and thereby extending the sensitivity in the low-frequency range, the output of GT3X + increased to a level, comparable to the AM7164. This attenuated the differences in the sedentary and lighter intensities in free-living.

This indicates that the lower output observed at the lower frequencies might not be due to lower sensitivity of the hardware in the newer generations of monitors per se but due to non-optimal matching of filtering and processing algorithms imbedded in GT firmware for mimicking the analogue AM7164 frequency and magnitude response. In addition, the old and newer generations differ in two important parameters that could influence the monitor output at a given frequency and amplitude; resolution of the A/D signal converters (8 bits vs. 12 bits) and sampling rate (10 Hz vs. 30 Hz).

### Strengths and limitations

The strength of the study is the combined use of mechanical setup with free living confirmation of the comparison. Furthermore, we used different batches of the same Actigraph generation and the newer activity monitors were randomly picked among more than 400 monitors compiled by three batches (bought at three different time points in the period 2009–2010). We also used multiple amplitudes of displacement within the range of displacement observed in human movement [27]. Taken together this increases the generalizability of our findings to other batches of Actigraphs and the performance within the displacement amplitude in humans.

The study also had some limitations. The activity monitors were acquired from different study centers and have been used by several different field investigators. Therefore we do not have complete knowledge of service history and frequency of use. To rule out batch differences we reran the protocol using six GT3X monitors randomly picked from an independent batch and six of the AM7164 used in the study. The results did not differ from the main results (data not shown). Previous observations have shown that the AM7164 output increases over time when used repeatedly in the field [16]. Thus, the difference between the newer and older generations could be a product of changes in the mechanical properties of the older accelerometer. This would probably not influence the shape of the response curves, as the observed drifting seems to be an offset drift (parallel shift of the curve) as Moeller et al. observed a constant increase at all settings in their setup (+2.5% over three months) [16]. This would consequently increase the bias observed in the low frequency range and decrease the bias in the medium frequency range. We only used the GT1M version 4 (bought in 2008), which might limit the generalizability of our finding to previous studies, as the signal processing is different in the newer generation (8-bit A/D signal converters in the older version vs. 12-bit A/D signal converters in the newer versions). However, our results are comparable to the findings by Rothney et al. [6], and smaller differences could be explained by differences in the mechanical setup.

Further, the free living study was based on a small sample. However, the study had time represented at all intensities of PA as would a background sample. Furthermore, we observed a significant difference between generations despite a small sample. Increasing the sample size would probably decrease the variance in the sample but not the mean bias. Post hoc inspections of Bland Altman plots revealed that the AM7164 output was higher in sixteen of twenty subjects whereas the bias was opposite (by ~10-20 CPM) for the remaining four subjects. There was no consistency in the position order of the activity monitors in the belt nor did we observe a different trend in the output of these monitors in the Actigraph calibrator compared to the remaining AM7164. It has been suggested that batch differences exist within the same brand of monitors [17]. The four monitors with the non-uniform direction of the bias were from the two different batches used in this study. Therefore we do not suspect this to explain the observation. As a mean CPM can be compiled through different intensity distributions, we investigated whether the four participants differed from the remaining sample herein. Interestingly, they spent ~40 min (p > 0.1) more time in the sedentary interval when assessed by the GT3X (LFE disabled), ~60 min more (p > 0.1) when assessed by the GT3X (LFE enabled) and ~80 min more (p < 0.05) when assessed by the AM7164 compared to the remaining sample. No substantial differences were observed in minutes spend at moderate and high intensities. This indicates that the four AM7164 monitors used for the participants with the non-uniform direction of the bias are less sensitive in the sedentary interval compared to the remaining sample. We therefore visually compared the trajectories of the AM7164 four monitors obtained in the mechanical setup to the remaining sample. When comparing the AM7164 on the short radius, the output from the four monitors was slightly lower than the remaining sample. This was not observed using the long radius, thus confirming that these four AM7164 monitors might in fact be less sensitive at the low intensities. No differences were observed when comparing the GT generations (data not shown). Larger inter-monitor variation has been observed between the AM7164 validated on the Actigraph calibrator compared to the newer GT-generations [6]. The non-uniform direction of the bias could thus be explained by the inter-monitor variation due to a lower sensitivity of the four monitors during sedentary activities compared the remaining batch. This problem would not be captured by the standard use of the Actigraph calibrator as the recommended gain limits are based on the peak value at 0.75 Hz. Even though we manually inspected the plots during calibration, we did not see a marked different output at the lower intensities when calibrating the four AM7164 monitors with specific monitors.

Finally, we scaled the one-minute cut-points to fit the ten-second epochs. This could compromise the precision of the cut-points related to the energy consumption. However, increasing the epoch time could blunt the response to especially higher PA intensities [28]. It would therefore an advantage to employ short epochs when evaluating the differences between generations at higher intensities.

## Conclusion and perspectives

In conclusion we observed significant differences throughout the frequency range between the older piezoelectric AM7164 and the newer MEMS-based GT1M, GT3X and GT3X + at two radii of oscillations on the vertical axis. Enabling the LFE did decrease the differences between the AM7164 and GT3X in the low frequency range, but increased the difference in the medium and higher frequencies. In free living enabling the LFE option attenuated the differences in mean PA completely, but induced a bias in the moderate PA intensities.

The observed differences in mean PA and across PA intensities would affect the calculated number of persons fulfilling the PA guidelines depending on the monitor generation used to collect the main bulk of data on which the guidelines are based. The differences could have potential implications when applying cut points derived from older generations to newer generations, due to intensity specific inter-generation differences in performance. Cut points for the newer generations should thus be based on validation studies using the newer generations. The bias could lead to misinterpretations of the observations when using multiple generations of Actigraph activity monitors.

## Abbreviations

PA: Physical activity; LFE: Low frequency extension; PA: Physical activity; CPM: Counts per minute; SEM: Standard error of the mean.

## Competing interests

The authors declare that they have no competing interests.

## Authors’ contributions

MRL, JCB, NCM, SB conception and design of the mechanical experiments; MRL, MG, BHH, and LBA conception and design of the free living study; MRL JCB and NCM performed experiments; MRL, JCB, and NCM analyzed data; MRL, JCB, NCM, MG, BHH, SB and LBA interpreted results of experiments; MRL prepared figures; MRL, JCB and NCM drafted manuscript; MRL, MG, BHH, SB and LBA edited and revised manuscript; MRL, JCB, SB, MG, BHH, LBA, and NCM approved final version of manuscript.
